# Cell Secretome Strategies for Controlled Drug Delivery and Wound-Healing Applications

**DOI:** 10.3390/polym14142929

**Published:** 2022-07-20

**Authors:** Ranya Ibrahim, Hillary Mndlovu, Pradeep Kumar, Samson A. Adeyemi, Yahya E. Choonara

**Affiliations:** Wits Advanced Drug Delivery Platform Research Unit, Department of Pharmacy and Pharmacology, School of Therapeutic Sciences, Faculty of Health Sciences, University of the Witwatersrand, 7 York Road, Parktown, Johannesburg 2193, South Africa; 2399287@students.wits.ac.za (R.I.); hillary.mndlovu@wits.ac.za (H.M.); pradeep.kumar@wits.ac.za (P.K.); samson.adeyemi@wits.ac.za (S.A.A.)

**Keywords:** secretome, biomaterials, wound healing, acellular medicine, tissue regeneration

## Abstract

There is significant interest in using stem cells in the management of cutaneous wounds. However, potential safety, efficacy, and cost problems associated with whole-cell transplantation hinder their clinical application. Secretome, a collective of mesenchymal stem-cell-stored paracrine factors, and immunomodulatory cytokines offer therapeutic potential as a cell-free therapy for the treatment of cutaneous wounds. This review explores the possibility of secretome as a treatment for cutaneous wounds and tissue regeneration. The review mainly focuses on in vitro and in vivo investigations that use biomaterials and secretome together to treat wounds, extend secretome retention, and control release to preserve their biological function. The approaches employed for the fabrication of biomaterials with condition media or extracellular vesicles are discussed to identify their future clinical application in wound treatment.

## 1. Introduction

The skin is considered the largest human organ that protects the outer body against the external environment. This includes physical, chemical, and microbial invasion, which lead to skin injury or trauma upon exposure [1,2,3]. Skin wounds and complicated wound-healing processes affect about one billion people worldwide and have an enormous influence on the human health care system, leading to an increase in financial cost [4,5]. Wounds are categorized as acute or chronic; acute wounds can heal quickly in a short period; however, if not treated properly, they can become chronic wounds [6,7].

Wound healing is an organized and highly regulated process that comprises the following phases; inflammation, proliferation, tissue remodeling, and extracellular matrix ECM deposition [5,8,9,10,11]. Acute wounds can be managed generally with a physiological wound-healing process. Chronic wounds can be managed with growth factors and cytokines, skin substitutes composed of polymeric materials and biologically derived substances to act as a structural support at the wound site; hyperbaric oxygen therapy (HBO_2_); and skin grafts [8,12]. Wound-healing management has been extending from traditional dressing to modern advanced types, which to some extent solve the problems associated with the traditional ones. Modern dressings must be biocompatible, biodegradable, and mimic the biological molecules involved in the body’s natural healing stages to provide greater adaptability to the wound bed, which is reflected in accelerated wound healing [6,13]. However, the approaches mentioned above have practical limitations as wound treatments [14].

New therapeutic approaches for the treatment of non-healing wounds have now been developed. One of the most promising approaches is using stem-cell-based therapy as an alternative approach for tissue repair and wound healing. Stem cells have a high capacity for self-renewal, interacting with the wound environment, and emitting bioactive secretions that accelerate wound healing. However, this technique has been faced with limitations such as biosafety, immune compatibility, potential tumorigenicity, infection risk, complicated material storage, and higher treatment costs [15,16,17,18]. Therefore, the development of more effective therapeutic strategies for advanced wound healing with minimized cost should be carried out.

Recent research proposed that it is worthwhile to use the paracrine activity of stem cells, where their secreted molecules yield higher therapeutic impacts than using cells. The secretome or condition media of stem cells play an essential role in regenerative medicine alternatives to living cells [16,19,20,21]. Secretome is defined as a range of bioactive molecules produced by a cell in the extracellular space, which includes but is not limited to proteins, nucleic acids, proteasomes, exosomes, microRNA, and membrane vesicles [22,23,24]. Secretome components are classified into the following: (a) a soluble portion comprising cytokines, chemokines, and immuno-modulatory molecules and growth factors; and (b) the extracellular vesicle, which is composed of microvesicles and exosomes that play an important role in cell–cell communication due to their involvement in microRNA and protein delivery [16,25,26].

Secretomes gained considerable attention in skin-wound management, as presented in vitro and in vivo studies that show the ability of secretomes to improve the wound-healing process by accelerating angiogenesis, inflammation reduction, and the stimulation of fibroblast and keratinocytes proliferation [27,28]. Secretome can exert the tissue-repair capability through different administration methods such as intravenous, intraperitoneal, or subcutaneous injection, either locally or systemically. However, these delivery methods can lead to rapid clearance. Biomaterials have been a promising approach to overcome the decreased retention time of secretome components in regenerative medicine. Enhanced bioavailability is reflected in a combination of secretome and biomaterials, which leads to increased therapeutic potential [29,30].

As an attempt to increase the retention time of secretome bioactive at the wound site, biocompatible biomaterials are used as carriers [31]. These biomaterials have the potential for re-epithelization and angiogenesis, decrease the possibility of infection after injury, and increase biocompatibility [32]. Both natural and synthetic polymers are widely used in regenerative medicine to deliver entrapped bioactive to tissues. They act as structural supports and controlled delivery systems [31,33]. This review provides insight into the combination of secretome with biomaterials for potential wound-healing applications. In addition, it highlights the approaches employed to fabricate biomaterials with condition media or extracellular vesicles to identify their future clinical applications in wound therapy.

## 2. Secretome

### 2.1. Secretome Composition

Secretome can be collected from a variety of human stem cell sources, with the most mentioned being umbilical cord tissue [34], bone marrow (BM-MSCs) [35], Wharton’s jelly mesenchymal stem cells (WJ-MSCs) [36], peripheral blood [37], adipose tissue (ASCs), placental tissues, and human umbilical cord perivascular cells (HUCPVCs) [38]. However, human adipose tissue-derived stem cells (ADSCs) gained a prime focus in tissue engineering due to their ease of separation and high harvesting rate [39]. ADSCs secretome has a great magnitude in regenerative medicine due to the positive effects of bioactive components in the treatment of cutaneous wounds, cardiovascular diseases, and CNS regeneration, amongst others [23]. ADSCs secretome can promote wound healing and accelerate wound closure via secreted growth factors [40,41].

**Figure 1:**
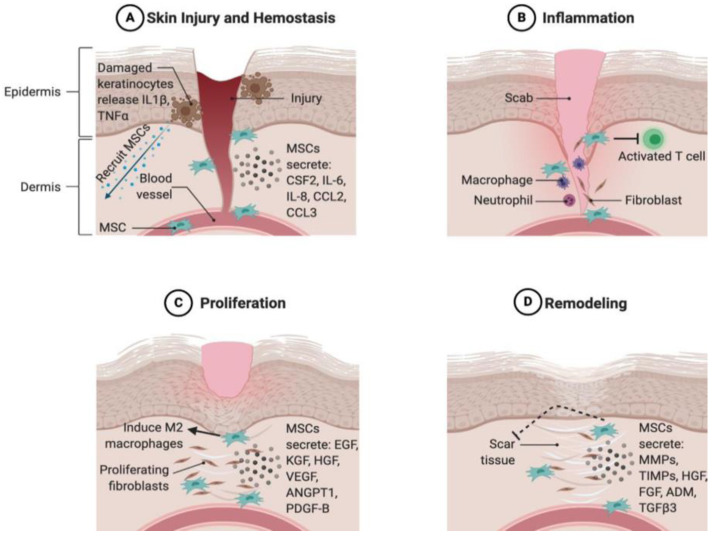
MSC recruitment to wounded skin and the inflammatory phase, and known and potential roles of MSCs in each phase of wound healing. (**A**) Skin injury and hemostasis. (**B**) Inflammation. (**C**) Proliferation. (**D**) Remodeling. Image reproduced with permission from Riedl et al. [44], Copyright 2021, Elsevier B.V. Ltd.

An analysis of secretome harvested from human adipose-tissue-derived mesenchymal stem cells confirmed the presence of increased levels of endothelial growth factor (EGF), hepatocyte growth factor (HGF), and basic fibroblast growth factor (bFGF). These proteins integrate with the cellular components of the dermis and facilitate the following processes: EGF promotes fibroblast migration and proliferation, HGF inhibits apoptosis, and bFGF promotes skin regeneration without fibrosis [42]. The secretome of human gingival fibroblasts revealed high amounts of pro-inflammatory cytokines such as IL-6, Arginase, MCP-1, and IL-8 [43]. HGF, FGF-2, VEGF, Ang-1, Ang-2, MMP-2, MMP-9, and TIMP-1 [43]. The cytokines revealed enhanced cutaneous wound healing of rapid re-epithelialization, decreased inflammation, angiogenesis promotion, and collagen deposition elevation, in addition to growth factors and ECM protein expressions [43]. The components of MSC secretome play an important role in wound-healing phases, as described in Figure 1 below.

### 2.2. Advantage of Secretome over Cell Therapy

Cell-based therapy has been applied for decades in regenerative medicine and tissue repair to treat different pathological conditions. Skin wounds are one of the cases that are treated with cell-based therapy; however, improved ones are required to overcome the wound problem worldwide. Cell-based skin substitutes as an example of cell-based-therapy-exerted positive results in accelerated wound closure with improved re-epithelization and vascularity [45,46]. However, they are very costly, require specific storage conditions, and cause the patient to become susceptible to infection and rejection [22]. Stem cell secretome has significant advantages over cell-based therapy, which circumvents living-cell-associated problems represented in tumorigenicity, infection transmission, and immune reactions [40].

Secretome can be produced according to the GMP-compliant process to be treated in the same manner as pharmaceutical agents, and this can be viewed as an additional advantage [40,47]. The use of a conditioned medium from human uterine cervical stem cells (CM-hUCESCs) for eye corneal ulcers in a lyophilized form gives a clear indication that secretome can be stored for an extended period without deterioration or loss of potency [48]. Mesenchymal stem-cell-conditioned media (MSC-CM) was implemented in bone regeneration rather than MSC and showed beneficial effects in avoiding the invasion-collection procedure of cells [49]. ADSC secretome produced by the maturation process could be helpful in the mass-production of secreted factors and account for a readily available supply of bioactive factors [40]. Secretome therapy’s cost-effectiveness can overcome the high cost of cellular therapy. The reduction of cell culture and immediate secretome therapies can be applied to manage acute pathological conditions such as military trauma, cerebral ischemia, and myocardial infarction, and modification of the biological product can take place to achieve a cell-specific effect [19,50].

Based on the advantages listed above, secretome has the potential to overcome the ethical problems associated with cellular transplantation. In addition to that, complications related to the survival and inaccurate differentiation of cells in the host tissue are reduced. The overall capability of cell therapy can be maintained by paracrine activity. Secretome-based therapies provide advantages such as availability, scalability, and longer shelf life [51]. In general, both cell-based therapy and secretome have advantages and disadvantages. However, the prolongation of the survival of transplanted cells and knowing how to predict decreased cell viability and biological functions during in vitro culture are the current challenges of cell-based therapies [40]. Accordingly, several strategies have been developed to improve the therapeutic efficacy of stem cells and secretome, such as genetic modification, preconditioning, and tissue engineering [40].

### 2.3. The Role of the Secretome in Different Stages of Wound Healing

Skin wound healing is a choreographed and closely regulated process comprised of inflammation, proliferation, matrix formation, and remodeling phases [52]. After skin injury, wound healing can be coordinated normally by keratinocytes, dermal fibroblasts, and immune cells [26,44]. However, secretome-based therapy has the potential to contribute to the acceleration of the wound-healing process. This is due to its components that promote anti-inflammatory factors, cell mitogenesis, re-epithelization, proliferation, and tissue remodeling, and induce neovascularization, leading to overall wound healing, particularly wound closure [42].

The secretome components relevant to various wound-healing stages include growth factors (PDGF, IGF-1, EGF, FGF, granulocyte-colony stimulating factor (G-CSF), GM-CSF, HGF, PGE2, TGF-βs, VEGF, and KGF), inflammatory proteins (IL-1, IL-8, IL-10, IL-6, tumor necrosis factor-alpha (TNF), leukemia inhibitory factor (LIF), IL-11, MCP-1, PGE2, IL-9, and IL-13), ECM proteins (MMP-1, MMP-2, MMP-3, MMP-7, TIMP-1, TIMP-2, ICAM, elastin, collagens, decorin, and laminin), and angiogenic factors (VEGF, ANG-1, ANG-2, PDGF, MCP-1, TGF-β1, FGF, EGF, CXCL5, MMPs, and TGF-α). The secretome effect on the inflammatory phase has been assessed by Lotfinia et al.; the report indicated the use of mesenchymal stem-cell-secretome to treat peripheral blood mononuclear cells in vitro [53]. The study found that pro-inflammatory cytokine production was reduced, while anti-inflammatory cytokine production increased [53]. Another study on mice excisional wounds injected with bone-marrow-derived stem cell secretome resulted in the promotion of wound healing by reduced inflammation mediated by macrophage polymerization [22].

A study by Park et al. indicated that secretome includes bioactive factors such as EGF, bFGF, and HGF, which are known to activate the PI3K/Akt and/or FAK/ERK1/2 signaling pathway [42]. This pathway is involved in the migration and proliferation of dermal cellular components during tissue repair. The bioactive and the activated pathway are believed to improve the proliferative and migratory capabilities of dermal fibroblasts, keratinocytes, and endothelial cells, among other biological components of the dermis [42].

In the proliferation phase of wound healing, soluble substances of the secretome can enhance fibroblast migration and the secretion of ECM components, particularly collagens I and III, resulting in wound-healing acceleration within the wound bed [42]. During the remodeling phase, the total collagen content increases, leading to wound contraction. This effect has been confirmed in a study that applied a human gingival fibroblast condition medium to treat wounds [43]. Endothelial cells treated with human multipotent adult progenitor cell-conditioned medium MAPC-CM also formed more vessel-like tubes [54]. The secretome accelerates wound healing by promoting angiogenesis. This has been demonstrated by a study carried out on wounds treated with MAPC-CM. The outcome of the study was an increasing number of endothelial cells and blood vessels in the wound bed due to increased VEGF in the CM, which accounts for a proangiogenic factor stimulating the vessel formation of endothelial cells [54]. Secretome components can accelerate wound healing by promoting target cell proliferation, differentiation, vascularization, and wound remodeling.

## 3. Secretome Applications in Wound Healing

Stem cell secretome or condition medium shows good outcomes in accelerating wound closure and promoting skin regeneration in wound healing. This evidence has been outlined in many studies due to secreted growth factors and cytokines and their potential for wound healing [22]. Many physiological processes relevant to wound healing are mediated by stem-cell-mediated paracrine and autocrine cell signaling pathways. Furthermore, the secretome is composed of several constituents with extensive regenerative potential for wound tissue.

**Figure 2:**
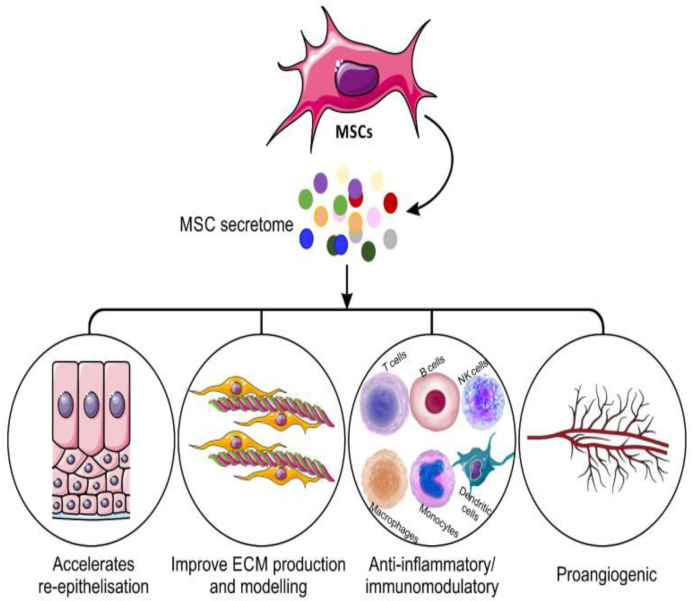
Mechanisms of mesenchymal stem cells secretome on wound healing. Image reproduced under an open access license from Ahangar et al. [22], Copyright 2020, © authors.

An analysis of the human adipose-derived stem cell secretome revealed a high level of various growth factors, as mentioned in Table 1. These biological factors play a crucial role in wound healing and tissue repair as they can promote skin tissue regeneration and modulate the immune response [55]. These secreted factors can act directly on normal wound-healing stages to promote re-epithelialization and angiogenesis and indirectly by immunomodulatory capacities. These factors can stimulate existing skin cells’ proliferative and migratory abilities through PI3K/Akt or FAK-ERK1/2, signaling an acceleration of wound healing [42]. The mechanisms of mesenchymal stem cell secretome in wound healing are illustrated in Figure 2. Therefore, extensive studies take place in this area to evaluate the different mechanisms for wound repair. Consequently, the focus is on the secretome of stem cells as a novel tool for treating various types of wounds. Current applications of the secretome from the various MSC sources, and their involvement in wound closure acceleration, are summarized in Table 1.

## 4. Secretome Delivery in Wound Healing

Biomaterials play an important role in tissue regeneration, which comprises delivering bioactives and provides structural support for endogenous cell invasion. For biomaterials to be applied, they must fulfill the following criteria involving biocompatibility, degradability, and suitable mechanical properties. Biomaterials are classified into three categories: naturally derived, synthetic, and chemically modified polymers. Natural biomaterials shown in this field comprise alginate, collagen, hyaluronan, and decellularized extracellular matrix (ECM). Biomaterial scaffolds made of synthetic polymers or ceramics such as polylactide-co-glycolide (PLGA) or beta-tricalcium phosphate (β-TCP) are extensively employed, with gelatin methacrylate (GelMA) being the natural material with chemical modifications [65].

Synthetic materials offer multiple advantages, such as cost, supply, and batch-to-batch homogeneity. However, they lack native tissue shape and structure. Hybrid hydrogels combining natural and synthetic materials have also been employed to attain the biological benefits of natural materials while attaining the benefits of tunable synthetic materials [66]. Biomaterials may be able to overcome the inadequate tissue retention of bolus EV and MSC-CM injections by offering a controlled release platform for healing tissues.

Biomaterials, which include scaffolds, meshes, matrices, hydrogels, and substrates, have completely transformed the way drugs are delivered and used. Some of the most frequently employed scaffolds are collagen-derived matrices, silk-based meshes/matrices, dextran hydrogels, and electrospun nanofiber matrices such as poly-L-lactic acid (PLLA) [67,68]. However, electrospun nanofiber matrices are recommended in biological applications. These scaffolds provide a three-dimensional (3D) structure that is similar to that of the extracellular matrix (ECM)-like nano-architecture [69]. These matrices have a similar tensile strength to skin, making them a suitable candidate for skin wound healing.

Biomedical hydrogels, which have a comparable structure to the natural ECM, have been highlighted as promising biomaterials for delivering therapeutics and cell components to wounds. The following characteristics should be present in an ideal wound-healing hydrogel scaffold: suitable mechanical qualities, good water retention, anti-infection capacity, injectable capacity, and excellent cell biocompatibility. Exosome-based administration via hydrogel, on the other hand, is likely to improve angiogenesis and tissue regeneration during wound healing [70]. Table 2 mentions some examples of biomaterials and their application in wound healing.

## 5. Structural Formulation Using Biomaterials with Secretome for Wound-Healing Applications

Polymer-based biomaterials are widely used in tissue engineering. They can mediate tissue engineering through their in vitro structural support to help cell–cell interaction and growth factors. They can aid in in vivo transplantation of the regenerated tissue to integrate structurally and functionally with the system [80]. Hydrogels, which are three-dimensional hydrophilic polymers, have been used as a bioactive scaffold material for drug delivery and cell encapsulation [80]. However, recent studies have identified that biocompatible hydrogels as carriers of MSC CM and MSC exosomes can maintain the bioactive molecules of the CM at the wound site [81]. This is an attempt to overcome cell-based therapy-associated risks in terms of lowering processing time and local storage conditions.

**Figure 3:**
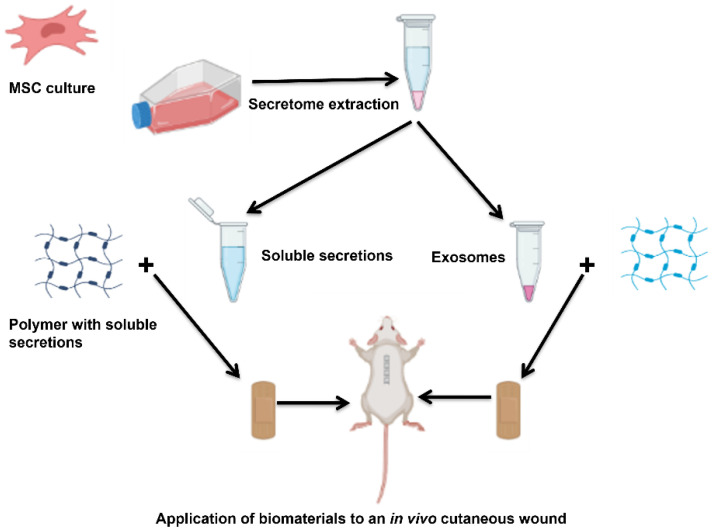
Schematic representation of MSC secretome extraction and exosome separation and combination with polymers for in vivo wound application.

MSc-secreted factors, which include extracellular vesicles and soluble factors, contribute mainly to their therapeutic benefit. However, the biomaterials can be combined with those factors, offering a delivery system to enhance the secretome retention rate and accelerate healing efficacy. This review highlights the use of biomaterials with secretomes in wound healing, providing insight into different examples applied in vitro and in vivo. Figure 3 below shows how secretome can be extracted from MSC and the CM and exosome mixed with polymers to develop a biomedical system that can be applied to treat in vivo wounds.

### 5.1. MSC Soluble Secretions and Their Combination with Biomaterials for Application in Different Wounds

Secretomes collected from in vitro culturing of MSC is also known as MSC-conditioned media (MSC-CM). The analysis showed the composition of the soluble factors, which are made up of cytokines, chemokines, growth factors, and hormones, with immunomodulatory, angiogenic, and anti-apoptotic functions [82]. The second part of secretion is termed extracellular vesicle secretions loaded with specific miRNA involved in both diagnosis and treatment [83]. The advantages of the in vitro applications of MSC-CM include cell proliferation and migration enhancement [84,85], the promotion of angiogenesis [85,86], and revealing anti-apoptotic and anti-inflammatory effects [84,87,88]. Furthermore, in vivo MSC-CM has demonstrated healing potentials in different wound types, which involve cutaneous wounds [89], burn wounds [73], and diabetic chronic wounds [59].

MSC CM can be administered by bolus injection, resulting in a shorter half-life and poor tissue retention. A combination of MSC CM with biomaterials presented a controlled release platform for healing tissues to overcome these adverse problems [65]. A recent study by Vasily et al. demonstrated the use of placental multipotent mesenchymal stromal cell (MMSC) secretome-loaded in chitosan hydrogel (MSC-Ch-gel) for infected burn wounds [90]. The method used in developing the MSC-CH gel involved the addition of chitosan solution to CM. The study revealed that MSC-Ch-gel had antimicrobial activity along with high anti-inflammatory abilities [90]. The high level of anti-inflammatory mediators was released upon the proteomic analysis of secretome besides proteins crucial for the different stages of wound healing. Furthermore, MSC-CH gel promoted skin tissue repair, which was observed after histological examination regarding higher vascularization and angiogenesis [90].

Another study conducted by HonorataK et al. evaluated the effect of human adipose tissue mesenchymal stem cell (HATMSC2) secretome-loaded hydrogel on chronic wounds [90]. The collagen hydrogel was prepared by adding the concentrated PBS to the type 1 collagen solution and then gently mixed. HATMSC supernatant was added to the collagen mixture before adding the crosslinker. The last step was adding 10K 4-arm Succinimidyl Glutarate PEG crosslinker followed by gentle mixing; then, the formed hydrogel was pipetted into Petri dishes and incubated at 37 °C for 1 h to allow for complete crosslinking [90]. The developed hydrogel was tested in an in vitro wound model using different cells, including endothelial, keratinocytes, and fibroblasts, during a 3-days culture. The results showed highly released interleukin-8 and macrophage chemoattractant protein-1 proteins from endothelial cells [91]. Additionally, pro-angiogenic activity was assessed using in vitro tube formation assay on human skin endothelial cells and confirmed by the expression of pro-angiogenic miRNAs, especially miR126, which shows the highest expression and antimicrobial activity against Staphylococcus aureus MRSA, and Pseudomonas aeruginosa was also confirmed [91].

A recent study developed by Victoria et al. focused on developing mesenchymal stem cell (MSC)-conditioned media (CM) loaded in hydrogel and its application in an in vitro hyperglycemic human dermal fibroblast to investigate the wound healing potential [92]. The components of the hydrogel were GelMA-PEGDA, loaded with MSC-CM, which demonstrated higher proliferation of the hyperglycemic fibroblast due to the combined effects of matrix properties together with the prolonged release of MSC-secreted bioactive molecules. Hence, it was potentially beneficial in diabetic chronic wounds [92].

A study by Anny et al. investigated the use of biocompatible polymers as transporters to preserve the bioactive molecules of CM at the wound site by combining MSC secretome with carrageenan and polyvinyl alcohol [31]. After preparing each hydrogel, the condition media embedded in each of it was polymerized, then it was derided and tested in in vitro human umbilical vein endothelial cells for angiogenic activity. Additionally, in in vivo application in mice, the cutaneous wound was carried, which showed the healing potential of both hydrogels’ impeded CM based on the proangiogenic properties of the secretome [31].

Another study applied BM-MSC secretome in vitro to primary cultured human corneal epithelial cells and an in vivo mouse model after both mechanical and alkaline corneal burn, hyaluronic acid (HA), and chondroitin sulfate (CS) gel were used as carriers (they were compared with secretome alone). The secretome was used in a lyophilized form to impart long stability and consistency to the different products. The study revealed secretome HA/CS gel accelerates epithelial wound closure after both injuries and can reduce neovascularization, scar formation, and hemorrhage after chemical injury [93]. Yiqing et al. developed a photo-crosslinking adhesive in situ-formed hyaluronic acid hydrogel grafted with the methacrylic anhydride and N-(2-aminoethyl)-4-[4-(hydroxymethyl)-2-methoxy-5-nitrophenoxy]-butanamide (NB) groups to encapsulate a lyophilized amnion-derived conditioned medium (AM-CM) [50]. The hydrogel displayed strong tissue adhesion, excellent mechanical properties, high elasticity, favorable biocompatibility, and prolonged AM-CM release. This was reflected in in vitro and in vivo accelerated diabetic wound healing resulting from the regulation of macrophage polarization and the promotion of angiogenesis [50]. Another study by Gabriella et al. developed a viscoelastic gel composed of hyaluronic acid (HA) and chondroitin sulfate (CS) to deliver lyophilized secretome from human bone-marrow-derived mesenchymal stem cells for the treatment of mechanical and chemical corneal injuries [93]. The in vitro and in vivo results accelerated epithelial wound closure and reduced corneal neovascularization, scar formation, and hemorrhage [93]. Vasily et al. developed placental multipotent mesenchymal stromal cell (MMSC) secretome-based chitosan hydrogel (MSC-Ch-gel) to treat infected burn wounds in rat [90]. Accelerated wound healing, tissue regeneration, reduced inflammation, improved re-epithelialization, and the encouragement of the development of well-vascularized granulation tissue were the outcomes [90]. The secretome produced by human fetal mesenchymal stem cells (hfMSC) in diabetic wounds was investigated by Bin Wang et al. [94]. The poly lactic-co-glycolic acid (PLGA)-encapsulating lyophilized hfMSC exhibited improved wound healing by encouraging vascularization and reducing inflammation in the cutaneous wound bed [94]. Chen et al. developed adipose-derived stem-cells-conditioned medium loaded in electrospun micro-nano fibers using poly lactic acid (PLA), which imparted protection and controlled release properties [95]. The in vitro and in vivo outcomes of the study were wound-healing acceleration and tissue regeneration [95].

### 5.2. MSC EVs and Their Combination with Biomaterials for Application in Different Wounds

Extracellular vesicles (EV) are nano or micro-sized vesicles that constitute the insoluble part of the secretome. They play a key role in cell-to-cell communication by transporting cargo directly into the cell or activating specified cell surface receptors. They are important in tissue repair and regeneration, disease detection, and oncology because they can transport membrane and cytosolic proteins, lipids, and RNAs [16,96]. Exosomes, the nano-sized vesicles, have become popular for application in cellular regenerative medicine, especially in wound healing. They organize cell-to-cell communication by carrying mRNA, miRNA, and proteins to target cells [70,97]. The following studies are examples demonstrating the combination of EV with biomaterials for wound healing. A study carried out by SHI-CONG TAO et al. describes the use of exosomes from microRNA-126-3p overexpressing synovium MSC mixed with chitosan hydrogel for cutaneous wound healing [96]. After the isolation and characterization of SMSC, the miRNA-126-3p lentiviral vector transfected them, then the exosomes were isolated and identified by specific procedures. After that, chitosan hydrogel-loaded exosome was prepared and tested in vitro and in vivo, which resulted in an in vitro promotion of proliferation and migration in human dermal microvascular endothelial cells (HMEC-1 cells) and human fibroblasts (FBs) [96]. However, a faster healing rate was reflected in diabetic wounded rats treated with CS-SMSC-126-Exos, which was reflected by epithelialization, granulation tissue formation, collagen deposition, and vascularization [96].

Another study demonstrated the preparation of chitosan/silk hydrogel sponge loaded with exosome derived from human gingival MSC and application to diabetic rat wounds. After the polymers dissolved, they stirred mechanically for 30 min. The hydrogel was prepared by the freeze-drying method and lyophilized to produce a sponge to which the collected exosomes were added [96]. Then, the hydrogel was applied to the wound area of the diabetic rats and accelerated wound healing. This is a noninvasive delivery system compared to the direct injection of exosomes, which can cause infection. The histological results showed enhanced re-epithelization, collagen deposing, neovascularization, and neuronal ingrowth [96].

An adipose-derived MSCs exosome loaded in alginate-based hydrogel has been applied to a full-thickness wound in a rat model. The study was performed by the isolation of ADSCs first, followed by exosome isolation and characterization; after that, the alginate hydrogel was prepared from alginates solution. The exosome was added and finally crosslinked with calcium chloride. The hydrogel was applied to assess its healing potential in a rat model. The exo-loaded hydrogel provided a novel delivery platform that accelerated wound closure by the enhancement of fibroblast migration, collagen synthesis, and vascularization [98]. A study done by Qijun Li et al. illustrated the dual-sensitive hydrogel comprised of poloxamer 407, and carboxymethyl chitosan encapsulates exosomes derived from human umbilical cord mesenchymal stem cells (hUCMSCs). The polymers were crosslinked with genipin, and the exosome suspension was mixed into the solution to form the hydrogel that exhibited sustained release behavior upon application to the cutaneous wound in a rat model, resulting in an enhancement of wound closure and tissue regeneration.

**Figure 4:**
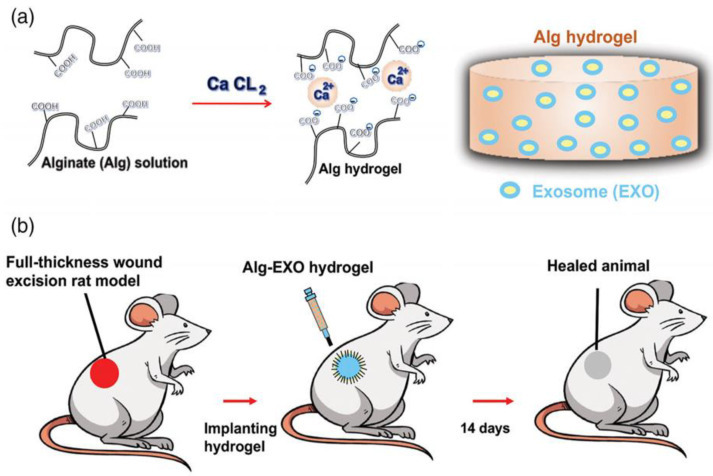
Schematic illustration of the hydrogel crosslinking and full-thickness wound excision mouse model used to evaluate the wound healing properties of alginate hydrogel-incorporated exosome (Alg-EXO). (**a**) Alginate solution loaded with adipose-derived stem cells (ADSCs)-derived EXOs cross-linked via ionic crosslinking. (**b**) Creation of a full-thickness wound excision rat model, and the transplantation of hydrogel into the injury area. Image reproduced with permission from Shafei et al. [98]. Copyright 2019, John Wiley and Sons.

In addition to that, the formation of skin appendages and the inhibition of inflammatory reactions [77] occurred. Wang et al. fabricated self-healing hydrogel from methylcellulose and chitosan via Schiff base reactions [99]. The hydrogel was loaded with exosomes extracted from placental mesenchymal stem cells. The hydrogel-loaded exosome exhibited accelerated wound healing, which was reflected in rapid wound contraction, new tissue formation, vascularization, and hair follicle and gland appearance when applied to the full-thickness wound in diabetic mice (Lepr^db^). Thus, wound healing promotion took advantage of an injectable hydrogel and the biocompatibility of the polymers [99]. Liu et al. explored the enhanced retention of adipose stem cell-derived exosome when combined with HA in the acute cutaneous wounds of nude mice [100]. The outcomes demonstrated that ASC-Exo+HA could significantly enhanced fibroblast activity, re-epithelialization, and vascularization in wound healing [100]. Figure 4 represents the hydrogel formation method using exosome-loaded polymers as one of the examples of the fabrication approach.

### 5.3. Secretome in 3D Bioprinting

Three-dimensional printing technology can be used for wound healing and skin engineering through the application of bioprintable materials known as bioinks. These bioinks must have good printability, mechanical stability, biocompatibility, biodegradability, non-toxicity, high availability, and high shape fidelity [101]. The 3D printing technology, rather than conventional approaches, can generate scaffolds that can resemble the complex ECM structures and provide a microenvironment for cell attachment, proliferation, distribution, and differentiation, with the capability to create functional tissue [102]. 3D technology can be used to carefully distribute cells, biological components, and growth factors into complex 3D bioscaffolds to construct tissue engineering structures that mimic biological ones. Leila et al. developed a collagen/alginate 3D bioprinted gel scaffold loaded with adipose-derived stem cells (ADSCs) for burn-wound healing, which resulted in complete epithelization and accelerated healing [103]. A study in bone regeneration used a 3D scaffold constructed from PCL and alginate hydrogel that contains lyosecretome (freeze-dried MSC secretome) for the controlled release of secretome to promote in vitro osteogenic differentiation [104]. Another study on 3D electrospun fiber scaffold, fabricated with polycaprolactone (PCL) and gelatin, was used as a cell culture medium with harvest (cell-free) MSC secretome, as well as continuous delivery from MSCs. The secretome was harvested and used to evaluate in vitro wound healing on corneal fibroblasts and subsequently explored a chemical burn on rabbit corneas employing an organ culture model. The outcome was epithelial layer recovery [105]. The effectiveness of 3D scaffold-based exosome treatment for skin regeneration has been examined in several research. Wang et al. verified that a biocompatible 3D porous self-healing methylcellulose-chitosan hydrogel, supplied with placental MSC-derived exosomes, promoted wound healing by cooperatively promoting angiogenesis and inhibiting apoptosis [106]. Therefore, using secretome 3D printing technology for wound healing is a promising area for further research.

## 6. Conclusions

Comprehensive studies have been done on the wound healing capability of MSC. They emphasized that their therapeutic benefit was mediated by paracrine secretions, including soluble factors and extracellular vesicle components collectively named secretome. They explore healing potential through the inhibition of apoptosis and inflammation, fibrosis, and angiogenesis. The secretome components can be delivered to the wound site when combined with biomaterials, which show better retention. Their effects proven in vitro and in vivo demonstrate valuable results in accelerating wound healing and promoting skin regeneration due to their tissue retention. To translate the experience of secretome to clinical situations, it is necessary to further understand its production procedures, which will reveal the way to enhance the production, advancement of isolation, and standardization methods for purification and characterization.

## 7. Future Prospective

Secretome-based therapeutics have become a potentially effective replacement for cell-based therapies. The secretome is at the vanguard of next-generation tissue and organ regenerative engineering applications due to its capacity to be produced, stored, and used as an off-the-shelf, ready-to-use product with minimal safety issues while maintaining the therapeutic benefits of stem cells. Advancing secretome-based therapeutics and determining their safety and efficacy will require the creation and evolution of methodologies and technology in MSC secretome culture, as well as a comprehensive grasp of secretome’s components. Biomaterials have also have been investigated as a supplement to control secretome production and as delivery systems. To accomplish clinical translation, the expansion of MSCs should be carried out under defined GMP culture conditions that are reproducible, scalable, and well-controlled, with the intention of limiting heterogeneity and enhancing the predictability of secretome-derived products in terms of composition and function.

## Figures and Tables

**Table 1:** The therapeutic outcomes of MSC secretome (MSC-S) in wound healing.

Stem Cell Type	Type of Wound and Model	Secretome Component	In Vitro Outcome	In Vivo Outcome	Ref.
Human (BMSC) from SCD patients	Murine excisional wound/endothelial cells in a mouse model	VEGF, IL8, MCP-1, and ANG	Using HUVECs in a 3-dimensional in vitro model demonstrates proliferation and migration in the presence of hypoxic CM that supports angiogenesis.	BMSC condition media exerts high trophic factors that promote angiogenesis and skin regeneration with accelerated wound healing.	[56]
ADMSC	Full-thickness skin excision on SD rats	VEGF	Rat dermal fibroblast cell line was treated with secretome revealed viability, proliferation ability, and higher migration capability, which represent better-wound healing. Macrophages were treated with secretome exert reduction of pro-inflammatory cytokines, including IL-6, TNF-α, and MCP-1.	Rapid wound closure enhanced fibroblast proliferation and migration. Moreover, the higher expression of VEGF promotes angiogenesis, which accelerates wound healing potential.	[8]
hUCESCs	Corneal epithelial cells/corneal ulcer on SD rats	TIMP-1, TIMP-2, FGF, and HGF	Enhanced epithelial wound healing, rapid regeneration, and the constitution of the corneal surface.	Bactericidal effect on corneal contact lenses (CLs) infected with *Escherichia coli* and *Staphylococcus epidermidis*.	[48]
hASC transfected with miR-146a	In vitro model using HUVECs	miR-146a UPA, (DPP IV), HGF, FGF-1, and FGF 2	the secretome146a promotes proliferation, migration, and tube formation of endothelial cells, reflected in enhanced proangiogenic properties. Additionally, the secretome miR-146a has immunomodulation effect that can potentially promote wound healing.	In vivo outcome was not studied.	[57]
ADSCs	6-mm diameter biopsy punch piercing in mice dorsal skin of male balb/c-nude mice	TGF-b1 and VEGF	Increased transdermal delivery of secretome proteins was expressed in an ex vivo porcine skin using iontophoresis as a permeation enhancer.	Acceleration of wound closure with reduced scars, represented by rapid re-epithelization, proliferation, increased tissue remodeling rate, and high vascularization.	[40]
HAFS	The full-thickness cutaneous excisional wound created on the dorsal skin of BALB/c mice	VEGF	In vitro effect was not tested in this study.	Speeding up of wound closure due to a decrease in myofibroblasts’ positive expression of α-SMA-rather than contraction enhanced re-epithelialization after 14 days of treatment, and overall fetal-like wound healing without scarring as a result of high expression of type III collagen accomplished by transformation of dermal fibroblasts into fetal-like fibroblasts rather than myelo fibroblasts.	[58]
HGFs	Dorsal excisional wounds of female BALB/c mice	IL-6, arginase, MCP-1, and IL-8 are examples of cytokines. Growth factors and ECM proteins such as HGF, FGF-2, VEGF, Ang-1, Ang-2, MMP-2, MMP-9, and TIMP-1 are also present.	Human keratinocytes and foreskin fibroblasts cells were used in vitro to evaluate a higher proliferation and migration rate. There was also an increase in capillary density, indicating enhanced angiogenesis. Additionally, increased collagen deposition is reflected in higher wound contraction without reducing fibrosis.	Wound closure acceleration with reduced inflammation, promotion of angiogenesis, and higher collagen deposition. Higher re epithelization.	[43]
Human bone marrow MSC	Full-skin thickness incision wound on the dorsal part of diabetic Wistar male rats (chronic diabetic wound)	bFGF and EGF expression	Human dermal fibroblasts cultured in a high glucose concentration medium resulted in an in vitro advanced wound closure due to rapid fibroblast migration, higher proliferation, and increased bFGF gene expression.	Acceleration of wound healing in terms of reduction of inflammation, increased vascularization, granulation tissue formation and enhanced, collagen deposition, and some trophic factor genes expression.	[59]
(WJ-MSCs)	Radiation-induced skin injury on Female Sprague–Dawley (SD) rats	------------	(HUVECs) growth rate and proliferation rate are increased. Enhanced number of blood vessels due to increased a-SMA expression.	Acceleration of wound closure enhances the quality of wound healing by promoting cell proliferation, sebaceous gland cell-like regeneration, and angiogenesis.	[60]
Gamma irradiation to induce apoptosis PBMCs	Burn wounds of 40 cm2 were created on the dorsum of the female Dan Bred pigs	IL-8 and VEGF	Histology studies carried out by using wound biopsies.	Improved epidermal regeneration and differentiation, a better wound quality without scarring, and increased numbers of CD31+ and ASMA+ cells as markers for angiogenesis.	[61]
MSC from fetal umbilical cord	Burn wound on the dorsal area of the Wister rat (Rattus Norvegicus)	bFGF	Histological analysis of skin tissues using M and H stains	Acceleration of wound closure, a more significant number of fibroblasts, high density of collagen fiber, and significant number of blood vessels.	[62]
Warton Jelly MSC	Burns on a 47-year-old woman’s left hand due to hot water exposure.	________	________	Three weeks of treatment with 10% secretome gel acceleration wound healing without scarring t	[63]
UMSC-Exos	Full-thickness skin wound on ICR mice and nude mice.	Exosome enriched microRNA represented as (miR-21, -23a, -125b and -145)	fibroblasts cells treated with recombinant TGF-b protein upon exposure to CM, leading to α-SMA suppression.	Wound healing promotion due to suppression of myofibroblast and scar formation through inhibition of transforming growth factor-b2/SMAD2 pathway.	[64]

**Table 2:** Biomaterials and their application in wound healing.

Polymer	Secretome Source	Bioactive Molecules	Type of Hydrogel	Biomedical Apps	References
Polyisocyanate (PIC)	Human adipose-derived stem cells (hASCs)	IL-10	Gel	Fibroblast wound healing assay or artificial wound	[71]
Carrageenan/poly(vinyl alcohol	SD-MSCs	VEGF	Hydrogel	full-thickness excisional wounds	[31]
Polycaprolactone/gelatin	Bone marrow-derived mononuclear cells	----------------	Electrospun scaffold	Diabetic wounds	[69]
Hyaluronic acid (HA) and chondroitin sulfate (CS)	Bone-marrow-derived human mesenchymal stem cells (hMSC)		Viscoelastic gel	Corneal wound	[72]
Methacrylate anhydride, Hyaluronic acid, N-(2-aminoethyl)-4-[4-(hydroxymethyl)-2-methoxy-5-nitrophenoxy]-butanamide (NB)	Amnion-derived conditioned medium (AM-CM)	VEGF and TGF-β1	In situ gel	In vivo diabetic wound	[50]
chitosan/collagen/*β*-glycerophosphate	Human umbilical cord mesenchymal stem cell		Thermosensitive hydrogel	In vivo burn wound	[50,73]
Pluronic F-127	human umbilical cord-derived MSC(hUCMSC)-derived exosomes	VEGF/(TGFβ-1)	A thermosensitive hydrogel	In vivo diabetic wound	[74]
Pluronic F127 /oxidative hyaluronic acid/(ε-poly-L-lysine, EPL)	Adipose mesenchymal stem cells (AMSCs)-derived exosomes	-------------	Hydrogel	Diabetic full-thickness cutaneous wounds	[70]
Polycaprolactone/gelatin	Bone-marrow-derived human mesenchymal stem cells	-------------	Electrospun fiber	In vitro corneal fibroblast cells and rabbit corneal organ culture system	[75]
Chitosan	Human endometrial stem cell (hEnSC)-derived exosome	-------------	Hydrogel	full-thickness cutaneous wounds	[76]
Carboxymethyl chitosan/poloxamer 407	Human umbilical cord-mesenchymal stem cells (hUCSCs)-derived exosomes	-------------	Thermo and pH-sensitive hydrogel	Rat cutaneous wound	[77]
Sodium Alginate/Sodium hyalurinate/PEG	Human BM-MSCs	VEGF and FGF	Hybrid gel	Tissue regeneration after surgry	[78]
Sodium alginate	Peripheral blood mononuclear cells (PBMCs)	CD31+ cells	NU-GEL™ Hydrogel	Burn wound	[61]
Chitosan/silk fibroin	Gingival mesenchymal stem cells (GMSCs) derived exosomes	Exosomal markers CD9 and CD81	Sponge	Diabetic rat cutaneous wound	[79]

## Data Availability

Not applicable.

## Abbreviations

Activated phosphatidylinositol 3 kinase/Protein kinase	PI3K/Akt
Adipose tissue-derived stem cells	ADSCs
Alginate hydrogel-incorporated exosome	Alg-EXO
Angiopoietin	Ang
Basic fibroblast growth factor	bFGF
Beta-tricalcium phosphate	β-TCP
Bone marrow mesenchymal stem cells	BM-MSCs
Chemokine	CXCL5
Conditioned medium from human uterine cervical stem cells	CM-hUCESCs
Endothelial growth factor	EGF
Extracellular matrix	ECM
Extracellular signal regulated kinase 1	ERK1
Extracellular vesicles	EV
Focal adhesion kinase	FAK
Gelatin methacrylate	GelMA
Good manufacturing practice	GMP
Granulocyte-colony stimulating factor	G-CSF
Hepatocyte growth factor	HGF
Human adipose tissue mesenchymal stem cell	
HATMSC	
Human bone marrow mesenchymal stem cell	BMSC
Human microvascular endothelial cells	HMEC
Human umbilical cord perivascular cells	HUCPVCs
Human umbilical vascular endothelial cells	HUVECs
Human uterine cervical stem cells	hUCESCs
Hyaluronic acid	HA
Hyperbaric oxygen therapy	HBO_2_
Interleukins	IL
Keratinocyte growth factor	KGF
Leukemia inhibitory factor	LIF
Matrix metalloproteinase	MMP
Mesenchymal stem-cell-conditioned media	MSC-CM
Mesenchymal stem cells	MSC
Mesenchymal stromal cell secretome-chitosan hydrogel	MSC-Ch
Monocyte chemoattractant protein	MCP
Multipotent adult progenitor cell-conditioned medium	MAPC-CM
Multipotent mesenchymal stromal cell	MMSC
Platelet-derived growth factor	PDGF
Polyisocyanate	PIC
Polylactide-co-glycolide	PLGA
Poly-L-lactic acid	PLLA
Sickle cell disease	SCD
Smooth muscle actin	SMA
Synovium mesenchymal stromal cell	SMSC
Tissue inhibitors of metalloproteinases	TIMP
Transforming growth factor	TGF
Tumour necrosis factor-alpha	TNF
Umbilical cord mesenchymal stem cells C-derived exosomes	UMSC-Exos
Vascular endothelial growth factor	VEGF
Wharton’s jelly mesenchymal stem cells	WJ-MSCs
Polyethylene glycol	PEG
